# Bilateral Peritonsillar Abscess in an Infant: An Unusual Presentation of Sore Throat

**DOI:** 10.1155/2017/4670152

**Published:** 2017-08-21

**Authors:** Mariana Manzoni Seerig, Letícia Chueiri, Janaina Jacques, Maria Fernanda Piccoli Cardoso de Mello, Martin Batista Coutinho da Silva, Daniel Buffon Zatt, Rosana Cristine Otero Cunha, Andre Souza de Albuquerque Maranhão

**Affiliations:** ^1^Department of Otolaryngology, Hospital Infantil Joana de Gusmão and Hospital Governador Celso Ramos, 152 Rui Barbosa St., 88025-301 Florianópolis, SC, Brazil; ^2^Department of Otolaryngology, Hospital Infantil Joana de Gusmão, 152 Rui Barbosa St., 88025-301 Florianópolis, SC, Brazil

## Abstract

**Introduction:**

Peritonsillar abscess is considered a suppurative complication of acute tonsillitis. It is usually unilateral and clinically evident bilateral presentation is uncommon. The condition affects mainly children older than 10 years and young adults. Herein we present a rare case of bilateral peritonsillar abscess in an infant.

**Presentation of Case:**

A 1-year-old boy presented with a two-day history of worsening sore throat, loss of appetite, vomiting, and fever. Examination of the oral cavity and oropharynx revealed enlarged and inflamed tonsils and a bilaterally congested and bulging soft palate. CT scan confirmed the hypothesis of bilateral peritonsillar abscess. Antibiotic therapy was instituted and after 5 days only slight regression of swelling of the soft palate was observed. He underwent a surgical procedure for draining the abscesses. After the procedure, he presented good clinical and laboratory evolution and was discharged home.

**Discussion:**

Although peritonsillar abscesses are considered common complications of acute tonsillitis bilateral cases are extremely rare, especially in early childhood. The diagnosis is based on history and physical examination and the treatment remains controversial among otolaryngologists.

**Conclusion:**

Bilateral peritonsillar abscess should be diagnosed and treated promptly and adequately to prevent respiratory obstruction and to avoid dissemination into the deep neck spaces.

## 1. Introduction

Peritonsillar abscesses (PTA) are collections of purulent material between the tonsil fibrous capsule and the pharyngeal constrictor muscles that usually develop near the superior pole [1, 2]. The formation of the PTA is probably an evolution from acute tonsillitis to peritonsillar tonsillitis to peritonsillar abscess [1]. Authors have proposed that drainage failure of suppurative inflammation by crypt blockage in acute tonsillitis leads to spreading the infection into the peritonsillar space. Others advocate that PTA formation may be a consequence of abscess in Weber's glands (salivary glands) located in the supratonsillar space [2]. Most cases are reported among older children, adolescents, and young adults and the diagnosis is made on the bases of the history and physical examination [3].

## 2. Presentation of Case

**Figure 1:**
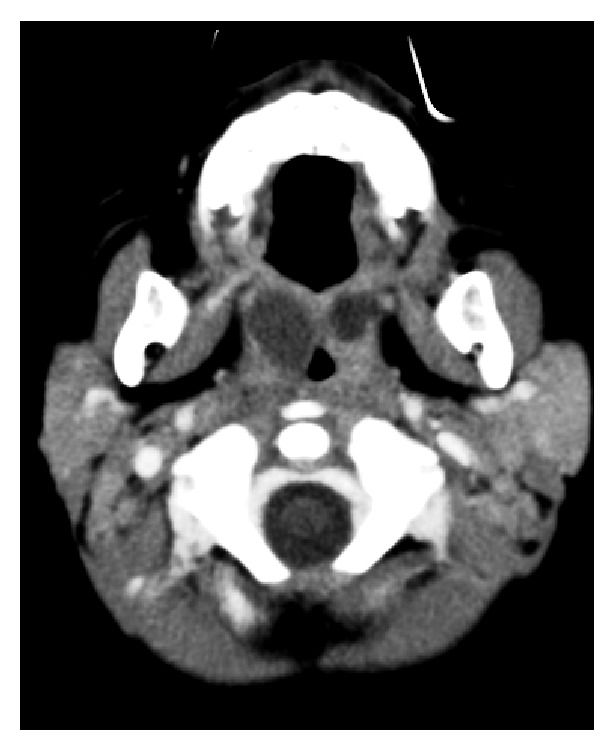
Contrast-enhanced computed tomographic scan, axial view. Enlargement of palatine tonsils and bilateral hypodense masses with thick rim enhancement. Slight obliteration of the parapharyngeal space.

**Figure 2:**
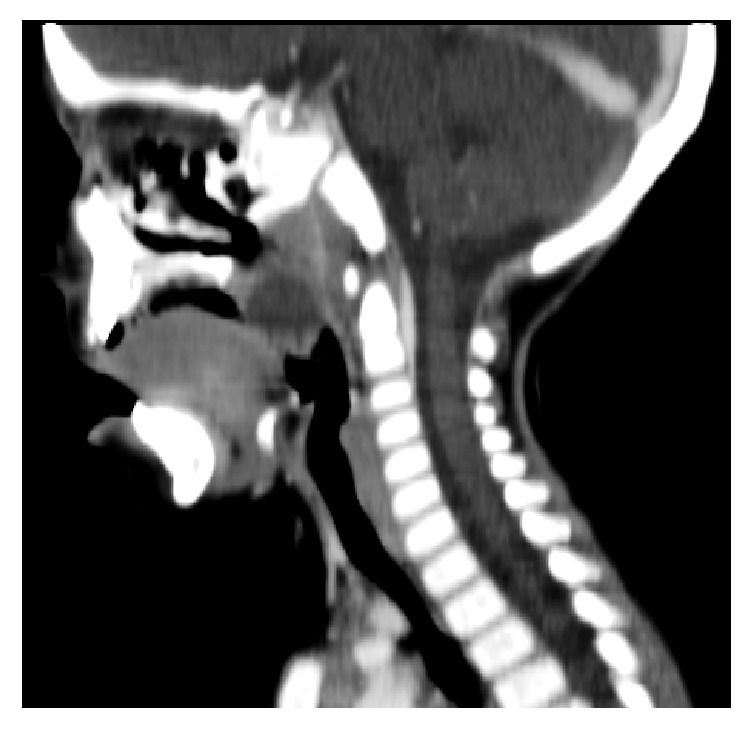
Sagittal view consistent with peritonsillar abscess.

A 1-year-old boy presented to the emergency department with a two-day history of worsening sore throat, loss of appetite, vomiting, difficulty with swallowing, and fever. On physical examination he had no fever and other vital signs were normal. Examination of the oral cavity and oropharynx revealed enlarged and inflamed tonsils and a bilaterally congested and bulging soft palate, more pronounced on the right, with a midline uvula. The laboratory test showed white cells count of 10.060/mm^3^ with predominance of lymphocytes (54.6%). The level of C-reactive protein was 93.1 mg/L. A monospot test was negative. Computerized tomography (CT) scan of neck showed bilateral hypodense masses, consistent with bilateral peritonsillar abscess (Figures 1 and 2). Conservative management with ceftriaxone (50 mg/kg/day), clindamycin (30 mg/kg/day), and prednisolone (1 mg/kg/day) was initiated. After 4 days of antibiotic therapy he remained afebrile but only slight regression of swelling of the soft palate was observed. He had another blood count test showing leukocytosis (17.330/mm^3^) even with predominance of lymphocytes. Due to therapeutic failure the ENT surgeon decided to submit him to a procedure for draining the abscesses. An incision was performed on superior pole of anterior tonsillar pillar bilaterally with drainage of purulent material. Tonsillectomy was not performed in this case. Cultures grew* Staphylococcus aureus* susceptible to ceftriaxone and resistant to clindamycin. He was discharged home two days after the procedure and must complete 10-day course of intravenous ceftriaxone. The patient's abscess resolved and there were no signs of recurrence at 12 months.

## 3. Discussion

Unilateral cases of PTA are extremely common occurring in about 30 persons per 100,000 per year thus accounting for approximately 45,000 cases per year in the United States [4–6]. In children the incidence of peritonsillar abscess is approximately 14 to 30 cases per 100,000 [7]. Schraff et al. reported a review of 83 patients with the diagnosis of unilateral peritonsillar abscess and found just two patients younger than 3 years, while Friedman et al. described a series of abscess in early childhood in which he found four patients with the same diagnosis in the same age group [8]. The actual frequency of bilateral peritonsillar abscess is not known; however, it has been seen at rates of 1.9% to 24% in reports of quinsy tonsillectomy (also known as acute abscess tonsillectomy), in which contralateral abscess is found at the procedure [5]. Most of the studies refer to adults and there are few case reports of bilateral peritonsillar abscess in children.

As tonsillitis is an infection that involves both tonsils, it is probable that progression to peritonsillar abscess also occurs bilaterally, showing different developmental stages on each side [1].

The correlation between peritonsillar abscess and immunocompromised hosts is not well described in the literature; however it is known that the immune status of children is different from that of adults. The compromised humoral immunity of these young patients allows opportunists to adhere to the host's tonsillar tissue and subsequently invade the epithelia, clinically seen as the progression of membranous tonsillitis to PTA. In addition, it is important to remember that patients who have immune dysfunction, diabetes mellitus, and HIV infection are at risk for atypical and more complicated cases of head and neck infections, so it is important to recognize these pathologies and start proper treatment earlier [9–11].

The vast majority (>70%) of peritonsillar abscess are polymicrobial and present aerobic and anaerobic organisms. The most common aerobes are* Streptococcus pyogenes* and* Streptococcus viridans*, while* Fusobacterium* and* Bacteroides* are the most common anaerobes [3, 5, 12].

The diagnosis of peritonsillar abscess is essentially clinical. PTA in general presents with a muffled voice, progressive odynophagia, difficulty in swallowing, referred otalgia, trismus, oral pooling of saliva, drooling, and fever. Unilateral cases exhibit asymmetry of tonsils and palate, uvula deviation, and unilateral otalgia. In case of bilateral abscess the difficulty of diagnosing stems from the fact that it does not demonstrate these classic signs [4, 5].

The differential diagnosis includes acute bacterial tonsillitis, infectious mononucleosis, and, less commonly, neoplasms such as lymphoma [8]. Besides the fact that diagnosis is eminently clinical, contrast-enhanced CT may help to differentiate bilateral PTA from other diseases and should be considered in patients who have trismus without unilateral inflammatory signs suggesting PTA. CT scan also helps in diagnosing complications, such as deep neck space infections [5].

An inadequately treated PTA can cause edema of the epiglottis or larynx or progress into parapharyngeal or retropharyngeal spaces reaching the mediastinum and causing a mediastinitis or it can spread upwards under the skull base. All of these conditions are potential life-threatening complications and must be recognized and addressed immediately [12]. The first and most important measure in treating PTA is securing of the airway [5]. The need for surgical drainage or needle aspiration in all cases of peritonsillar abscess remains controversial. Antibiotic therapy alone is an option for those without airway compromise. Kim et al. found that younger age, fewer episodes of acute tonsillitis, and small abscess pockets are significant predictors of good response to nonsurgical treatment in pediatric peritonsillar abscess patients. This study also shows that the response of nonsurgical treatment significantly deteriorates after 7.5 years of age suggesting that pediatricians should consider nonsurgical treatment for young child [7]. Surgical options for management of PTA are incision and drainage or quinsy tonsillectomy. Some authors advocate that quinsy tonsillectomy is the most definitive procedure and should be done if a young child is being taken to the operating room. Otherwise it is known that there is an increased risk of postoperative hemorrhage in patients undergoing quinsy tonsillectomy. Qureshi et al. observed a changing trend in surgical management of PTA with significant decrease in the rate of tonsillectomies and significant increase in the rate of incision and drainage procedures [13].

Whereas the patient is only 1 year old and it was impossible to perform the drainage without general anesthesia, we opted for trying intravenous antibiotic therapy. He had no fever during the hospitalization and showed improvement of oral intake but after four days of conservative treatment we observed worsening of infectious parameters in laboratory tests and the physical examination remained unchanged. Then we decided to submit him to a surgical procedure for drainage. We opted for incision and drainage instead of tonsillectomy because of the young age of the patient, no history of recurrent sore throat, and the high risk of bleeding on quinsy tonsillectomy. After one-year follow-up he did not present another episode of tonsillitis.
